# Enhancing autophagy mitigates LPS-induced neuroinflammation by inhibiting microglial M1 polarization and neuronophagocytosis

**DOI:** 10.3389/fncel.2025.1546848

**Published:** 2025-02-20

**Authors:** Jingjing Guo, Yun Li, Kun Ma, Guohai Su

**Affiliations:** ^1^Jinan Central Hospital, Cheeloo College of Medicine, Shandong University, Jinan, China; ^2^Department of General Medicine, Central Hospital Affiliated to Shandong First Medical University, Jinan, China; ^3^Department of Pediatrics, The First Affiliated Hospital of Shandong First Medical University and Shandong Provincial Qianfoshan Hospital, Jinan, China; ^4^Department of Cardiology, Central Hospital Affiliated to Shandong First Medical University, Jinan, China

**Keywords:** autophagy, LPS, NF-κB, microglia cells, cell phenotype

## Abstract

**Background:**

Autophagy, a regulator of inflammation, has been implicated in various central nervous system pathologies. Despite this, the role and mechanisms of autophagy in lipopolysaccharide (LPS)-induced neuroinflammation are not clear. This study investigated whether autophagy can play a neuroprotective role in LPS-induced neuroinflammation.

**Methods:**

Primary microglial cells and male C57BL/6 J mice were treated with LPS, autophagy inhibitors (3-methyladenine, 3-MA), or autophagy activators (rapamycin). Cell viability, NF-κB pathway activation, pro-inflammatory cytokine expression, M1 polarization, autophagy markers, and neuronal damage were evaluated via various techniques including CCK-8 assay, Western blot analysis, ELISA, immunohistochemistry, and histological staining.

**Results:**

LPS (1 μg/mL) effectively inhibited cell viability, stimulated the expression of IκB-α and NF-κB, and simultaneously suppressed autophagy protein expression. The pro-inflammatory cytokines IL-1β and IL-6 showed a significant increase. Contrary to the effect of 3-MA, the rapamycin treatment inhibited the polarization of microglia cells to the M1 type in the various groups of microglia cells after LPS stimulation. This was evidenced by decreased expression of cytokines IL-1β, IL-6, and CD86, and increased expression of Arg-1, IL-10, and CD206. *In vivo* experiments found that mice with injections of LPS and 3-MA in the lateral ventricle showed significantly increased expression of IκB-α and NF-κB in brain tissues, elevated levels of pro-inflammatory cytokines, decreased autophagy levels, and increased necrotic neurons. There was increased aggregation of microglia cells and increased neuronophagocytosis. Conversely, mice injected with rapamycin showed enhanced neuronal cell autophagy, decreased expression of pro-inflammatory cytokines and apoptosis, and reduced neuronophagocytosis.

**Conclusion:**

Enhancing autophagy can effectively mitigate LPS-induced neuroinflammation by inhibiting microglial M1 polarization and neuronophagocytosis, thereby protecting neuronal integrity. These findings suggest potential therapeutic strategies targeting autophagy in neuroinflammatory conditions.

## 1 Introduction

Autophagy has multitiered immunological functions and can regulate infection, inflammation, and immunity (Deretic et al., 2013). It plays an important role in the innate immune response against pathogen infection (Xiao and Cai, 2020). It has been confirmed that autophagy has a protective effect on nervous system infections caused by various pathogens, and targeting intracellular pathogens through macroautophagy/autophagy is considered to be an important part of host innate immunity (Aikawa et al., 2018). Activation of autophagy can provide necessary cell components and ATP for the activities of macrophages and enhance the function of phagocytes. In addition, some genes of autophagy and macrophage phagocytosis are shared, such as Beclin1 (a key regulator in autophagy), Vps34 (vacuolar protein sorting 34, a class III phosphatidylinositol 3-kinase), and autophagy-related gene 5 (involved in autophagosome formation), etc. (Ogawa et al., 2018). Therefore, autophagy genes play an important role in managing the bactericidal function of macrophages.

Microglia are the resident macrophages in the central nervous system, which are crucial for the normal development and homeostasis of the brain. They can not only eliminate pathogens invading the nervous system but also play a role in removing senescent cells and harmful substances (Xu et al., 2016; Fricker et al., 2018). By releasing inflammatory mediators and neurotrophic factors, microglia induce phagocytosis of cell fragments and apoptotic cells and degradation of microorganisms (Saijo and Glass, 2011). These capabilities enable microglia to respond to central nervous system injuries, facilitating injury repair and restoring intracerebral balance (Hanisch and Kettenmann, 2007). Additionally, during infection of the central nervous system, inhibition of autophagy can induce the neuroinflammatory response of microglia, thus damaging the nervous system (Lee et al., 2019). It has been shown that microglia overactivation and release of pro-inflammatory cytokines (such as IL-1β, TNF-α, and IL-6) play a key role in neuroinflammation (Haque et al., 2020). However, activated microglia secrete various pro-inflammatory factors and inflammatory mediators in pathological injury or diseases with developmental defects, which are harmful to nearby neurons (Gupta et al., 2018).

In particular, bacterial infections have been recognized as a major trigger for immune responses, often leading to the release of lipopolysaccharides (LPS), which are toxic components derived from the outer membrane of Gram-negative bacteria. LPS acts as a potent pro-inflammatory agent by interacting with toll-like receptor 4, activating downstream signaling cascades, including the nuclear factor kappa-light-chain-enhancer of activated B cells (NF-κB) pathway, thereby mediating immune and inflammatory responses (Ha et al., 2008). During bacterial infections, the release of LPS can lead to the activation and polarization of microglia in the central nervous system. Activated microglia can adopt either M1 or M2 phenotypes, with M1 polarization being associated with pro-inflammatory cytokine release and neuroinflammation (Tang and Le, 2016). Studies have shown that microglia activated by LPS can release many cytotoxic molecules such as inflammatory factors (Tang and Le, 2016; Huang et al., 2017; Wen et al., 2022). Furthermore, LPS can induce autophagy in microglia, macrophages, hepatocytes, and peritoneal mesothelial cells (Waltz et al., 2011; Wang et al., 2013; Chen et al., 2014). In addition, LPS can significantly reduce the expression of Vps34 in N9 microglia by activating the P13KI/AKT/mTOR pathway, but the decrease of Vps34 level prevents the maturation of autophagosomes (Ye et al., 2020). Specifically, LPS exposure has been shown to stimulate the production of various pro-inflammatory cytokines, such as L-1β and IL-6, thereby contributing to neuroinflammatory cascades and neuronal damage (Wen et al., 2022). Understanding the nuances of how bacterial infection, through LPS release, influences microglial polarization within the central nervous system is critical for developing therapeutic interventions.

Autophagy plays a crucial role in regulating inflammation by interacting with innate immune signaling pathways, eliminating endogenous inflammasome agonists, and influencing the secretion of immune mediators. Induction of autophagy can play a neuroprotective role (Wang et al., 2012). Given that LPS stimulates neuroinflammation and consequent neuronal injury, we wonder whether autophagy can play a neuroprotective role in this process. To investigate this query, this study assessed the role of autophagy in LPS-induced neuroinflammation. Both *in vivo* and *in vitro* experiments demonstrated that modulating autophagy inhibited LPS-induced M1 polarization in microglial cells, thereby ameliorating neuronophagocytosis and protecting neurons. Our findings suggest that enhancing autophagy may serve as a promising therapeutic strategy for the management of neuroinflammatory conditions and the protection of neuronal integrity.

## 2 Materials and methods

### 2.1 Study animals

Male C57BL/6 J mice (6–8-week-old; *n* = 30) were purchased from the Central Experimental Laboratory of the School of Medicine, Shandong University. They were maintained in an environment with a temperature range of 22°C to 26°C, a daily temperature variation not exceeding 3°C, and a relative humidity level of 50–60%. All experimental procedures were approved by the Ethics Committee of the First Affiliated Hospital of Shandong First Medical University (approval no. [2022] No. S013).

### 2.2 Isolation and culture of primary microglial cells

After sacrificing six mice, the brain tissues were carefully dissected and the cortical tissues were isolated. After removal of blood vessels, meninges, and other tissues, the collected cortical tissue was cut into small pieces and were then subjected to digestion with a solution containing 0.25% trypsin and DNA enzyme at 37°C for 10 min, which was conducted for four cycles. After digestion, the samples were filtered through a 200-mesh sieve (Hyclone, Logan, UT, United States) to eliminate undigested tissue fragments, resulting in a single-cell suspension. The suspension was then centrifuged to pellet the cells, which were resuspended in DMEM medium (Hyclone) containing 20% fetal bovine serum (Gibco, Grand Island, NY, United States), 0.1 mg/mL of streptomycin, and 100 U/mL of penicillin (Hyclone). After allowing the cells to adhere to the culture surface, the non-adherent cells and debris were removed by washing three times with phosphate-buffered saline (PBS) devoid of Ca^2+^ and Mg^2+^. Finally, to achieve further purification of the microglial cells, adherent cells were treated with 0.8 g/L trypsin–EDTA and incubated at 37°C until the upper layer of cells began to exhibit significant retraction and detachment. After washing with PBS, the remaining adherent cells were collected, representing the purified microglial population. They were then cultured in the complete DMEM medium at 37°C with 5% CO2.

### 2.3 Cell treatment and grouping

The cells were treated with different concentrations of LPS (0.5 μg/mL, 1 μg/mL, and 2 μg/mL), to determine the optimal concentration. According to different treatments, the cells were divided into the control group, LPS (1 μg/mL), 3-methyladenine (3-MA) + LPS (1 μg/mL), and rapamycin (RAPA) + LPS (1 μg/mL). Specifically, the primary microglial cells in the RAPA+LPS and 3-MA + LPS groups were treated with autophagy regulators RAPA (1 μM) and 3-MA (5 mM) for 2 h, respectively. Microglial cells in each group were then co-cultured with LPS (1 μg/mL) for 24 h. The cells in the control group were untreated.

### 2.4 Cell counting kit-8 assay

The toxic effect of LPS on primary microglial cells was determined using a cell counting kit-8 (CCK-8) assay kit (Hanbio, Changsha, China). Briefly, cells were seeded in a 96-well plate at a density of 10^5^ cells per well and incubated overnight at 37°C. After treatment with various concentrations (0.5 μg/mL, 1 μg/mL, and 2 μg/mL) of LPS (Sigma, St. Louis, MO, United States) for 24 h, the microglial cells were resuspended in 100 μL serum-free DMEM and 10 μL CCK-8, and incubated at 37°C for 4 h. The optical density at 450 nm (OD450) was measured using a microplate reader. Cell viability was calculated using the formula: (OD450 of the experimental group – OD450 of the blank control group) / (OD450 of the control group – OD450 of the blank control group) × 100%. The cell viability was normalized to that of the control group.

### 2.5 Immunofluorescence

The microglia cultured on the slides in each experimental group were fixed with 4% paraformaldehyde at room temperature for 15 min, rinsed thrice with PBS, and subsequently incubated in 5% normal goat serum and 0.05% Tween-20 for 1 h. Next, the slides were treated with the anti-LC3 antibody (Proteintech, Wuhan, China) at room temperature for 1 h. After rinsing thrice with PBS, the slides were exposed to a fluorescent secondary antibody and then incubated for 1 h. The samples were analyzed using a fluorescence microscope.

### 2.6 Animal treatment and grouping

Male mice were randomly divided into the control, LPS, LPS +3-MA, and LPS + RAPA groups, with 6 mice in each group. Anesthesia was induced using pentobarbital sodium (0.75 mg/10 g). After disinfection, the anterior fontanelle was exposed. The coordinates for the injection site in the lateral ventricle were set as follows: the anterior–posterior coordinate was +0.5 mm, the left–right coordinate was −1.0 mm, and the dorsoventral coordinate was −1.8 mm. Drug administration was performed through a drilled hole at the specified coordinates. All drugs were injected at the same location using the same method. The dosage of LPS was 0.5 mg/kg, of 3-MA (Sigma) was 1 mg/kg, and of RAPA was 10 μg/kg. After injection, the skin was disinfected and sutured.

### 2.7 Sampling

Twenty-four hours post-injection, the mice were sacrificed and the brain tissues were collected. The frontal and temporal lobe tissues near the injection site of the lateral ventricle, as well as hippocampal tissues, were used for Western blot and immunohistochemistry analyses. The remaining brain tissues were homogenized and lysed.

### 2.8 Western blot analysis

Primary microglia and brain tissues were collected after treatment and subjected to extraction of proteins on ice using RIPA lysis buffer and a protease inhibitor. Protein concentration was determined using the bicinchoninic acid method. After heating at 95°C for 5 min, approximately 40 μg of protein was loaded into each well of the vertical electrophoresis chamber. Subsequently, a 10% SDS-polyacrylamide gel electrophoresis was performed to separate protein samples, which were then transferred to a PVDF membrane. The membrane was blocked with 5% skimmed milk for 1 h, followed by overnight incubation with primary antibodies, including rabbit anti-mouse LC3 antibody (Proteintech), rabbit anti-mouse Beclin-1 antibody (Affinity, United States), rabbit anti-rat inhibitor of NF-κB (IκB)-α antibody (Merck, NJ, United States), rabbit anti-rat NF-κB antibody (Merck), rabbit anti-rat p-IκB-α antibody (Merck), rabbit anti-rat p-NF-κB antibody (Merck), CD86 (Wuhan USCN Sciences Co., Ltd., China), CD206 (Wuhan USCN Sciences), and tubulin (Hyclone) at 4°C. The membrane was washed with Tris-buffered saline with Tween for 5 min, repeated 3 times, then incubated with secondary antibodies of Goat anti-rabbit HRP antibody (Merck) and Goat anti-mouse HRP antibody (Merck) for 1 h. After washing, color development was conducted using enhanced chemiluminescence. Relative protein expression was determined using gray values.

### 2.9 Elisa

Primary microglia cells and brain tissues were lysed after treatment, and the supernatant was extracted. The levels of IL-1β, IL-6, IL-10, and Arg-1 in the supernatant were measured with corresponding ELISA kits (MultiSciences, Hangzhou, China), as per instructions. After incubation and washing, the substrate was added and incubated for 30 min in the dark. The absorbances were measured on a microplate reader.

### 2.10 Immunohistochemistry

The brain tissues were processed using standard techniques to obtain paraffin-embedded sections with a thickness of 5 μm. After deparaffinization, antigen activity was revived using microwave repair. Subsequently, the sections underwent three washes in PBS, followed by a 10-min incubation in a 3% hydrogen peroxide solution. The sections were then blocked with 5% BSA for 1 h. The mouse anti-CD68 antibody (Merk) was applied drop by drop and incubated overnight at 4°C. This was followed by three washes in PBS and incubation with the goat anti-mouse secondary antibody (Merk) at 37°C for 30 min. After washing, the sections were subjected to color development with 3,3′-Diaminobenzidine at room temperature for 5 min, with a subsequent hematoxylin counterstaining for 5 min. Finally, the sections were dehydrated, mounted, and examined under a microscope. Three independent fields of view were selected under 400× magnification and the number of CD68-positive cells within each field was counted. The average value of CD68-positive cells was compared among groups.

### 2.11 Nissl staining

The brain tissues were prepared into paraffin sections with a thickness of 4 μm. Deparaffinization was carried out routinely, followed by a 10-min treatment with the Nissl body staining solution (Hyclone), differentiation with 95% ethanol, and sealing with neutral resin. Subsequently, the samples were examined under a microscope, and images were acquired and analyzed. Each slice from each group was photographed at three randomly selected fields of view at 400× magnification. Within each field of view, necrotic neurons were selected as a standard reference for positive cell counting. The total number of necrotic neurons within the entire image was quantified, and the average was calculated.

### 2.12 Hematoxylin and eosin staining

The brain sections were deparaffinized with xylene I and II for 10 min each, followed by immersion in water and sequential exposure to 100, 90, 80, and 70% ethanol for 5 min each, with subsequent rinsing in running water. Subsequently, the sections were stained with hematoxylin and eosin for 5 min, followed by rinsing in running water. Next, differentiation was carried out using 5% acetic acid for 1 min, followed by rinsing in running water. Counterstaining with eosin was performed for 1 min, followed by rinsing in running water. Dehydration steps involved sequential immersion in 70, 80, 90, and 100% ethanol for 10 s each. After treatment with xylene for 1 min, the sections were mounted and observed under a microscope. The hematoxylin and eosin (H&E) staining images were assessed by an independent pathologist from a third party. In each group, three random fields of view at 400× magnification were selected for each slice. The focal cell aggregates, i.e., the distinct nodular structures, were defined as microglial nodules. The number of microglial nodules was counted within each field of view, and the average was calculated.

### 2.13 Statistical analysis

Each experiment was conducted at least three times, and the data were presented as mean ± standard deviation. Student’s *t*-test was used for comparing data between two groups, while one-way analysis of variance was data comparison across multiple groups. A *p*-value of less than 0.05 indicates statistical significance.

## 3 Results

### 3.1 Effect of LPS on the viability of microglial cells

**Figure 1:**
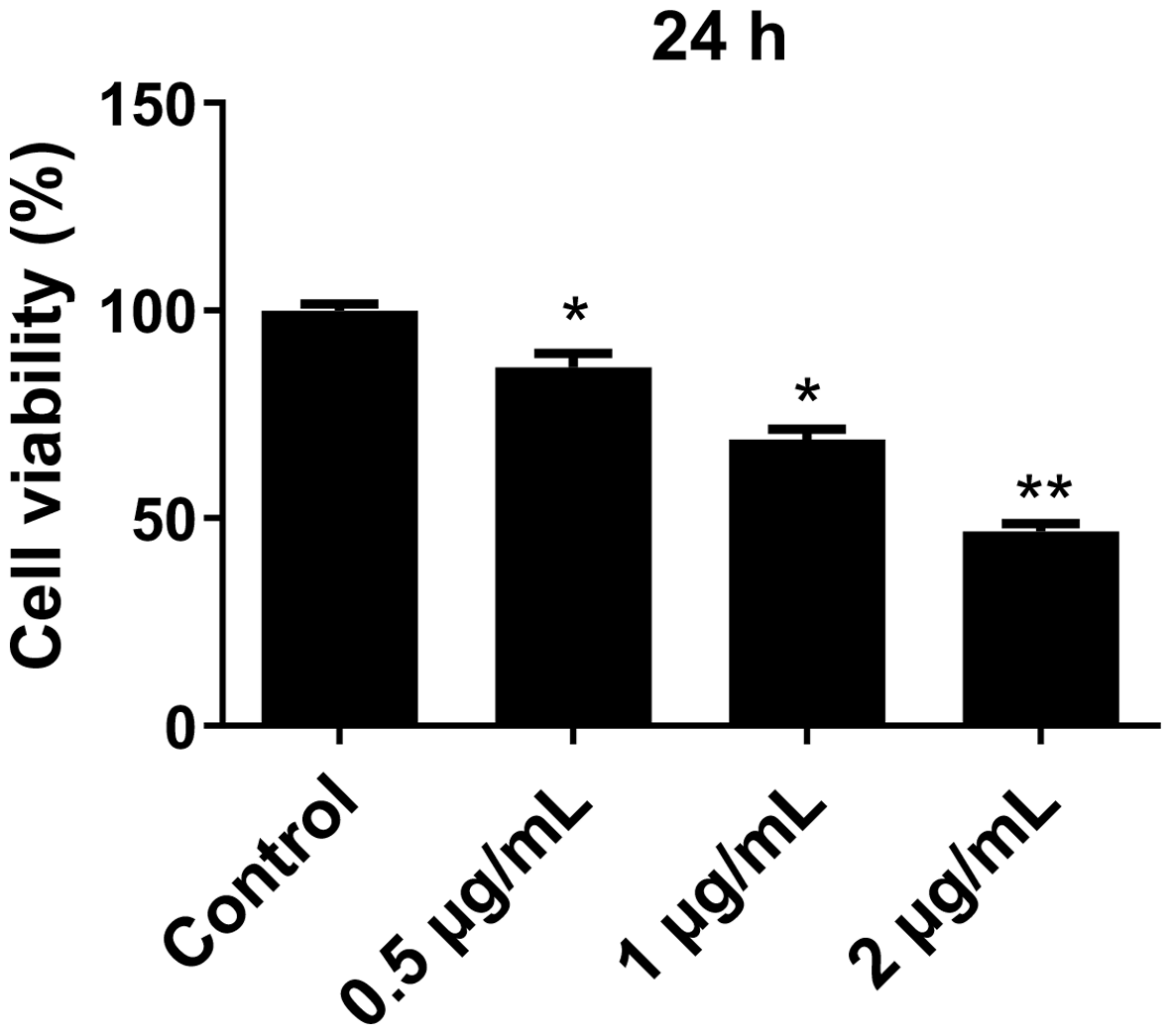
Effect of different concentrations of LPS on the viability of primary microglia cells. Microglia cells were treated with different concentrations of LPS for 24 h, and cell viability was determined using the CCK-8 assay. The cell viability was normalized to that of the control group. All data are presented as the mean ± standard deviation; *n* = 3; *p < 0.05; **p < 0.01 vs. control.

Primary microglial cells were exposed to different concentrations of LPS (0.5 μg/mL, 1 μg/mL, and 2 μg/mL) for a duration of 24 h. The CCK-8 assay was used to assess cell viability. The results revealed that post-cryopreservation and resuscitation, the microglial cells in each group maintained consistently low levels of viability. After treatment with LPS for 24 h, the cell viability of the LPS-treated groups was significantly lower than that in the control group (*p* < 0.05) (Figure 1). When LPS concentration reached 2 μg/mL, there was a notable reduction in cell viability (*p* < 0.01), indicating a substantial increase in cell death. Consequently, we selected a concentration of 1 μg/mL LPS for subsequent experiments.

### 3.2 Effect of LPS on the NF-κB pathway and the expression of pro-inflammatory factors in primary microglial cells

**Figure 2:**
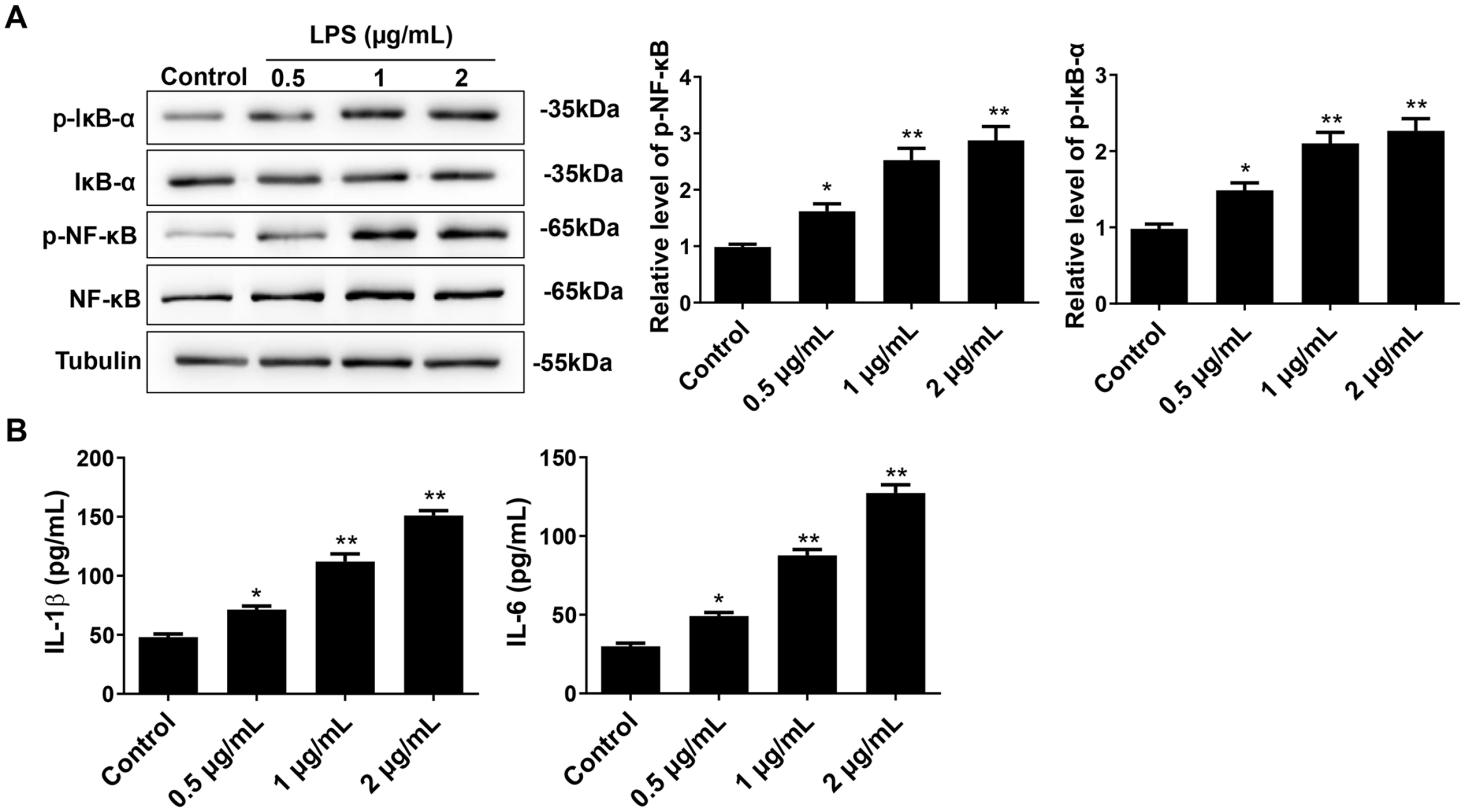
Effect of different concentrations of LPS on NF-κB pathway and pro-inflammatory cytokines in primary microglial cells. **(A)** After 24 h of cell intervention with different concentrations of LPS, the NF-κB, IκB-α, p-NF-κB, and p-IκB-α expressions were detected by Western blot. **(B)** The pro-inflammatory cytokines IL-6 and IL-1β were detected by ELISA. All data are presented as the mean ± standard deviation; *n* = 3; *p < 0.05; **p < 0.01 vs. control.

Western blot was used to detect NF-κB/IκB-α expression, while ELISA was performed to quantify the levels of M1-type pro-inflammatory factors IL-6 and IL-1β. The results showed that the p-NF-κB and p-IκB-α expression increased after LPS treatment in a dose-dependent manner (Figure 2A). Notably, the levels of p-NF-κB and p-IκB-α in cells treated with 0.5 μg/mL, 1 μg/mL, and 2 μg/mL of LPS were significantly higher than those in the control group (*p* < 0.05). However, there was no significant difference between 1 μg/mL and 2 μg/mL of LPS (*p* > 0.05). Furthermore, the expression of pro-inflammatory factors IL-6 and IL-1β also significantly increased with the increase of LPS concentration (Figure 2B). Compared to the control group, the levels of IL-6 and IL-1β after treatment with 0.5 μg/mL, 1 μg/mL, and 2 μg/mL of LPS were significantly increased (*p* < 0.05). Therefore, these results suggest that LPS may induce the activation of the NF-κB/IκB-α pathway in microglial cells, leading to the release of pro-inflammatory factors.

### 3.3 Enhanced autophagy suppresses the activation of NF-κB pathway after LPS treatment in microglial cells

**Figure 3:**
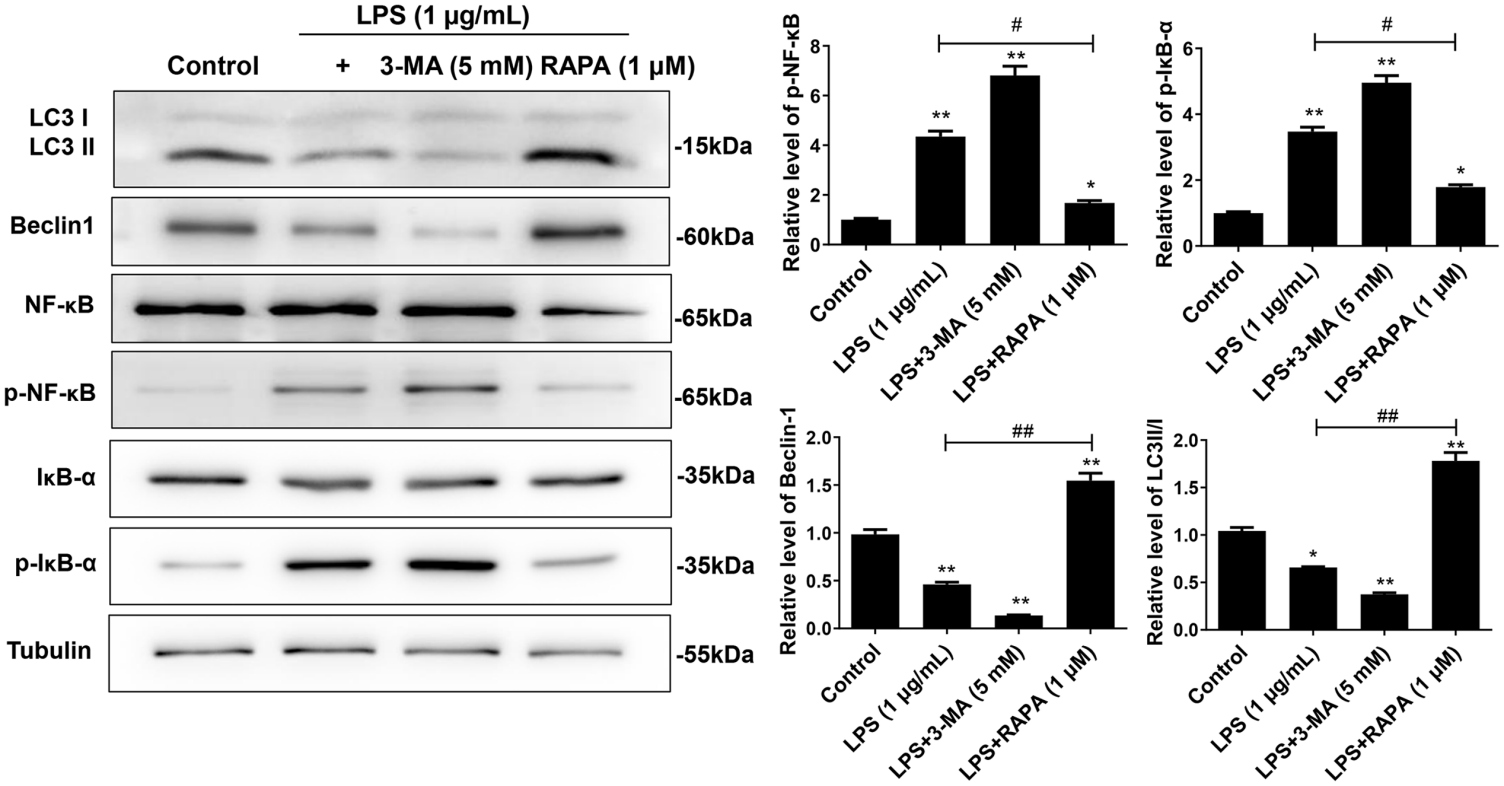
Effect of autophagy on the expression of NF-κB pathway proteins in microglial cells. Primary microglial cells intervened by RAPA and 3-MA were co-cultured with LPS for 24 h. Expression levels of LC3II/I, Beclin-1, p-NF-κB, p-IκB-α, NF-κB, and IκB-α were detected by Western blot analysis. All data are presented as the mean ± standard deviation; *n* = 3; *p < 0.05; **p < 0.01 vs. control. ^#^*p* < 0.05, ^##^*p* < 0.01.

To observe the effect of autophagy on the NF-κB pathway in microglial cells after LPS stimulation, Western blot was used to detect the protein expression of LC3II/I, Beclin-1, NF-κB, IκB-α, p-NF-κB, and p-IκB-α (Figure 3). The results showed that compared to the control group, the expression of p-NF-κB and p-IκB-α was significantly increased in the LPS and LPS + 3-MA groups, while the expression of autophagy proteins LC3II/I and Beclin-1 was inhibited (*p* < 0.05), indicating that inhibiting autophagy may promote the expression of the key proteins in the NF-κB pathway after LPS treatment. The LPS + RAPA group had significantly enhanced expression of autophagy proteins but significantly inhibited expression of p-NF-κB and p-IκB-α compared with the LPS group (*p* < 0.05), indicating that enhancing autophagy may inhibit the activation of the NF-κB pathway after LPS treatment.

### 3.4 Enhanced autophagy inhibits the M1 polarization of microglia and the release of pro-inflammatory factors induced by LPS

**Figure 4:**
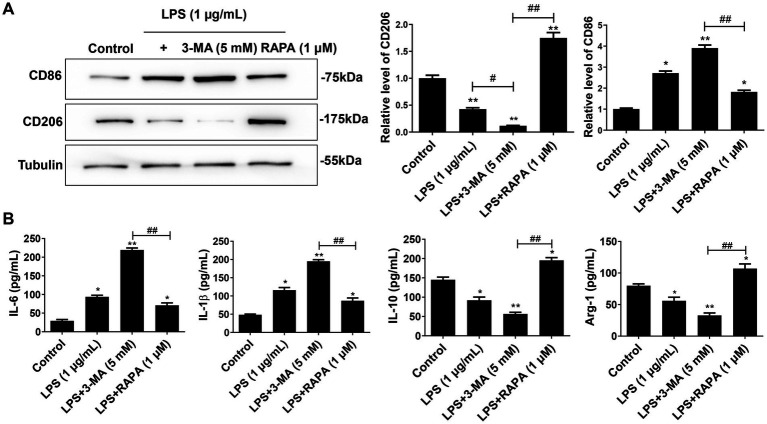
Autophagy inhibits M1 polarization of microglia. Primary microglial cells pre-treated with RAPA and 3-MA were co-cultured with LPS for 24 h. **(A)** The expression of CD86 and CD206 was detected using Western blot. **(B)** The levels of IL-1β, IL-6, IL-10, and Arg-1 were measured with ELISA. All data are presented as the mean ± standard deviation; *n* = 3; *p < 0.05; **p < 0.01 vs. control. ^#^*p* < 0.05, ^##^*p* < 0.01.

To determine the effect of autophagy on LPS-induced polarization of microglia, we measured the expression levels of M1 markers (IL-1β, IL-6, and CD86) and M2 markers (IL-10, Arg-1, and CD206) after treatment with 3-MA or RAPA. Western blot and ELISA results showed that compared to the control group, both LPS and LPS + 3-MA groups promoted M1 polarization, as evidenced by the enhanced expression of CD86 (Figure 4A), IL-1β, and IL-6 (Figure 4B) (*p* < 0.05). In contrast, RAPA inhibited M1 polarization compared to the LPS group, leading to a significant decrease in the expression of CD86, IL-1β, and IL-6, while enhancing M2 polarization with significantly increased expression of CD206, IL-10, and Arg-1 (*p* < 0.05). These results suggest that enhanced autophagy suppresses M1 polarization and the release of pro-inflammatory factors.

### 3.5 Enhancing autophagy inhibits the expression of NF-κB pathway proteins in mouse brain and microglial cells

**Figure 5:**
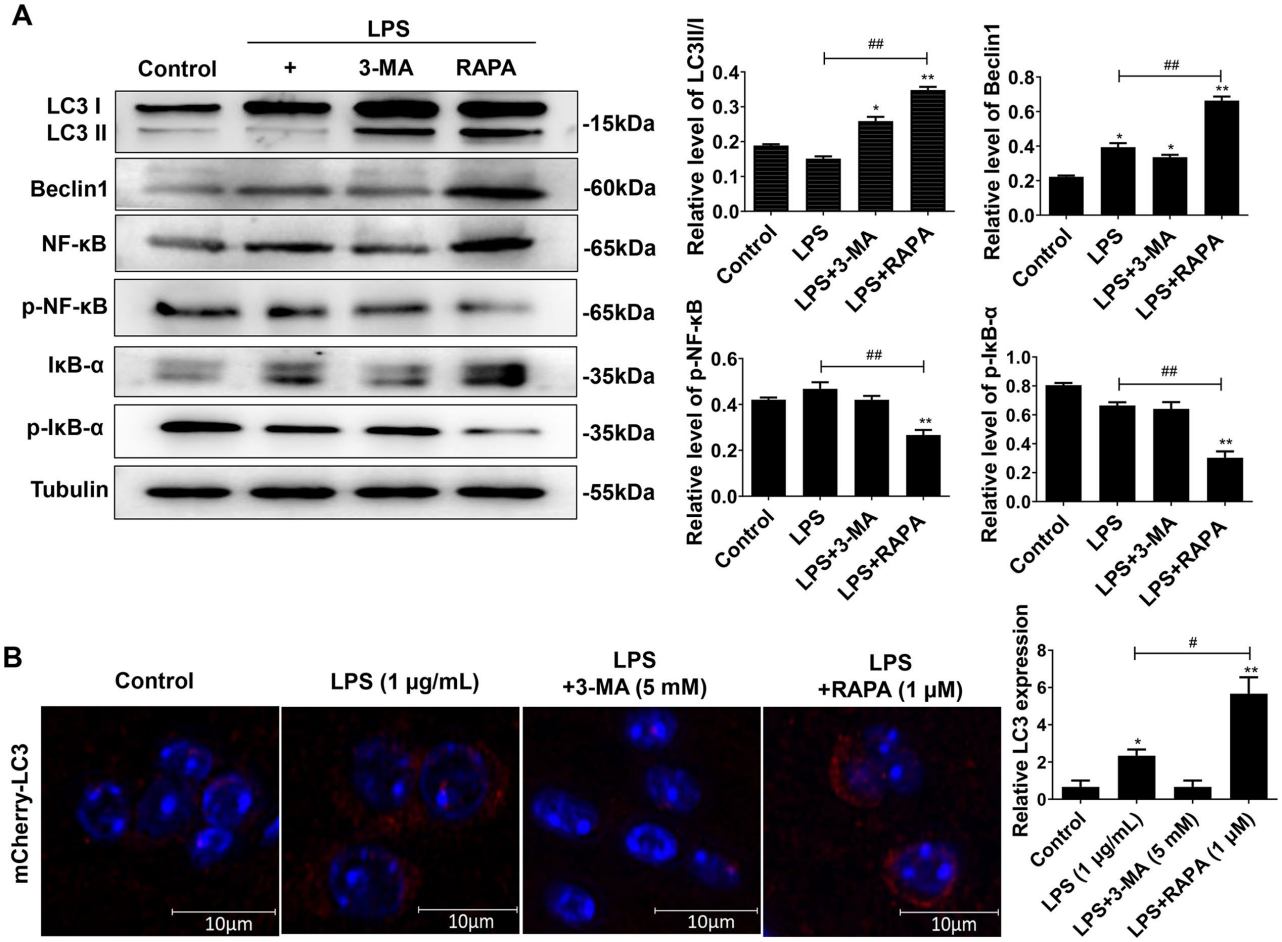
Effect of autophagy on the expression of NF-κB pathway proteins in mouse brain tissues as well as on the expression of LC3 in microglial cells. The 3-MA, RAPA, and LPS were stereotactically injected into the mouse brain. After 24 h, the frontal and temporal lobe tissues near the injection site of the lateral ventricle, as well as hippocampal tissues, were collected for Western blot analysis. **(A)** The expression levels of LC3II/I, Beclin-1, p-NF-κB, p-IκB-α, NF-κB, and IκB-α were detected by Western blot. **(B)** Immunofluorescence was used to evaluate LC3 expression in microglial cells. Scale bar: 10 μm. All data are presented as the mean ± standard deviation; *n* = 3; *p < 0.05; **p < 0.01 vs. control. ^#^*p* < 0.05, ^##^*p* < 0.01.

We further verified the effect of autophagy on the NF-κB pathway after injection of LPS, 3-MA, and RAPA in the lateral ventricle of mice. The expression of LC3II/I, Beclin-1, p-NF-κB, p-IκB-α, NF-κB, and IκB-α were detected using Western blot (Figure 5A). Moreover, autophagy intensity was assessed in microglial cells using immunofluorescence (Figure 5B). The results revealed that compared to the control group, there was no significant difference in the expression of p-NF-κB and p-IκB-α in the LPS and LPS + 3-MA groups (*p* > 0.05), but the expression of autophagy proteins LC3II/I and Beclin-1 significantly decreased (*p* < 0.05). In comparison to the LPS group, the RAPA treatment significantly promoted the expression of autophagy proteins while inhibiting the expression of p-NF-κB and p-IκB-α (*p* < 0.05). Furthermore, immunofluorescence analysis showed that compared to the control and LPS groups, RAPA significantly enhanced the expression of LC3 (*p* < 0.05). However, LC3 expression did not significantly change in the LPS + 3-MA group (*p* < 0.05). Therefore, enhancing autophagy *in vivo* can inhibit the expression of NF-κB pathway proteins, thereby suppressing its activation.

### 3.6 Autophagy inhibits M1 polarization and reduces neuronophagocytosis of microglial cells in mouse brain tissues

**Figure 6:**
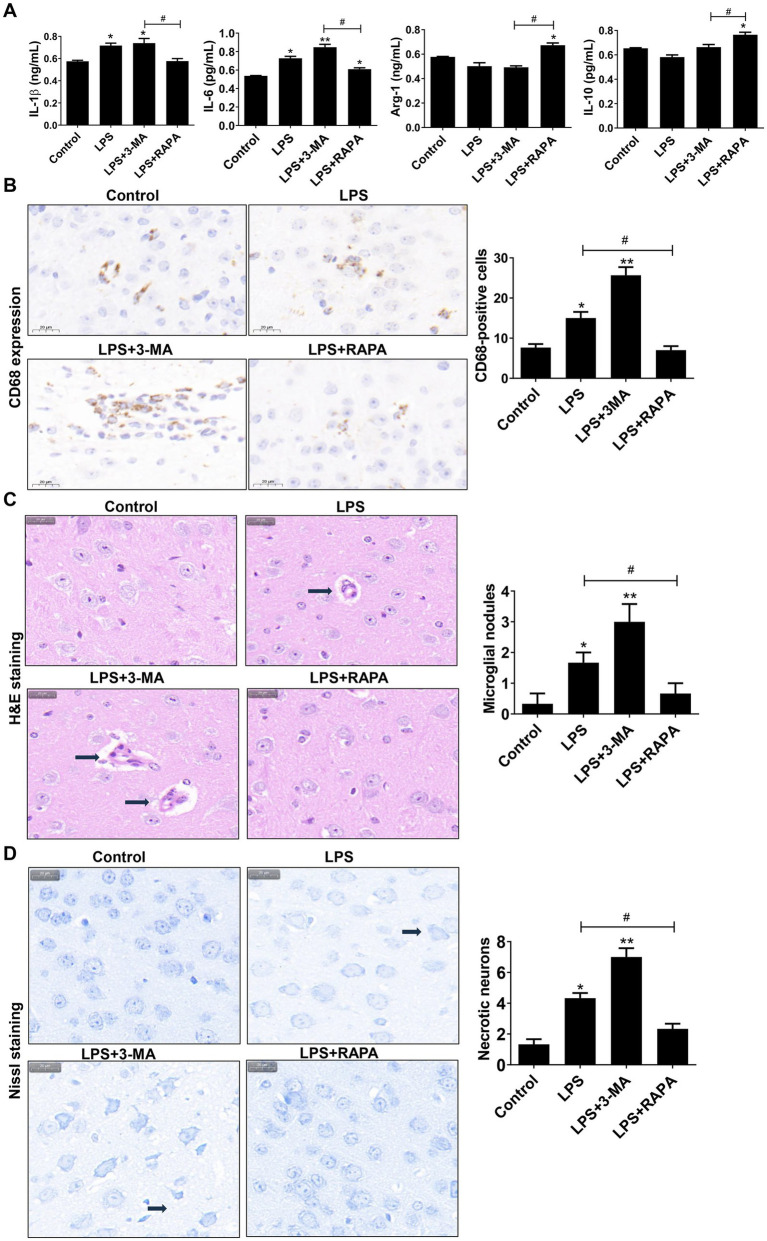
Autophagy regulates neuronal cell polarization and neuronophagocytosis in mouse brain tissues. **(A)** ELISA was used to detect the levels of IL-6, IL-1β, Arg1, and IL-10. **(B)** Immunohistochemistry was used to stain CD68, which was used to label microglial cells and observe microglial cell aggregation. The number of CD68-positive cells is compared among groups. **(C)** H&E staining was used to observe microglial nodules (arrows). The number of microglial nodules in each group is presented. **(D)** Nissl staining was used to assess necrotic neurons. Scale bar: 20 μm. The number of necrotic neurons in each group is presented. All data are presented as the mean ± standard deviation; *n* = 3; *p < 0.05; **p < 0.01 vs. control. ^#^*p* < 0.05, ^##^*p* < 0.01.

We also evaluated the effect of autophagy on M1 polarization *in vivo* by detecting polarization factors (IL-1β, IL-6, IL-10, and Arg-1). ELISA showed that the expression levels of pro-inflammatory factors IL-1β and IL-6 were significantly higher in the LPS and LPS + 3-MA groups than in the control group (*p* < 0.05) (Figure 6A). Compared with the LPS + 3-MA group, the LPS + RAPA group had significantly decreased IL-1β and IL-6 levels but significantly increased anti-inflammatory factors IL-10 and Arg-1 (*p* < 0.05). On the contrary, there was no significant change in IL-10 and Arg-1 levels of the LPS and LPS + 3-MA groups (*p* > 0.05).

Meanwhile, the CD68 expression on microglial cells was assessed using immunohistochemistry. As shown in Figure 6B, there was high CD68 expression in the LPS and LPS + 3-MA groups, indicating the activation of microglial cells. Moreover, there was a clustering of activated microglia, accompanied by considerable neuronal cell rupture and tissue destruction. Such observations were not noted in the LPS + RAPA group. Statistical analysis revealed a significant increase in CD68-positive cells in both the LPS group and the LPS + 3-MA group compared to the control group (*p* < 0.05). In contrast, the LPS + RAPA group showed no significant difference in the number of CD68-positive cells compared to the control group (*p* > 0.05). Notably, there were significant differences between the LPS group and the LPS + RAPA group (*p* < 0.05). Consistent with the immunohistochemistry results, the H&E staining results showed that the number of microglial nodules in the LPS group and LPS + 3-MA group significantly increased than that in the control group (*p* < 0.05) (Figure 6C), suggesting significant activation and aggregation of microglial cells and increased neuronophagocytosis. Additionally, the Nissl staining results also revealed cell structure damage in the LPS and LPS + 3-MA groups, with a significant increase in necrotic neurons (*p* < 0.05) (Figure 6D). However, the LPS + RAPA group exhibited inhibition of microglial cell aggregation. Compared with the LPS group, the number of microglial cells and necrotic neurons significantly decreased (*p* < 0.05). The results indicate that enhancing autophagy can inhibit LPS-induced M1 polarization and reduce neuronophagocytosis of microglial cells.

## 4 Discussion

Autophagy serves as a dual-sided mechanism in cellular processes. Under mild physiological stress, autophagy typically acts protectively within cells. Nonetheless, disruptions in autophagic pathways or excessive autophagic activity may result in cell death (Wang et al., 2012; Liu et al., 2023). Autophagy is intricately involved in the pathogenesis and progression of various diseases arising from pathogenic agents, often affecting pivotal organs such as the central nervous system, liver, kidneys, and heart in the human body (Wang et al., 2024). At different stages of disease development or under specific intervention conditions, specific molecules can determine whether autophagy plays a protective or cytotoxic role (Barnard et al., 2016; Cooper, 2018; Jung et al., 2020; Meyer et al., 2021). There is still controversy as to whether autophagy plays a neuroprotective role or promotes cell death in ischemic brain injury. Studies have reported that in ischemic stroke, upregulation of autophagy exerts a neuroprotective effect while blocking autophagy leads to rapid neuronal necrosis progression (Carloni et al., 2008; Rami and Kogel, 2008). Conversely, conflicting reports suggest detrimental implications of autophagy in ischemic stroke (Qin et al., 2010; Zhang et al., 2010). This study examined the effect of LPS-induced neuroinflammation on autophagy and M1 polarization of microglia through both *in vivo* and *in vitro* experiments. Additionally, the study also investigated the influence of autophagic levels on neuronophagocytosis using various autophagy modulators.

Different pathological factors may have varying effects on autophagy. Previous studies (Arroyo et al., 2013; Ma et al., 2020) have found that peptidoglycan can activate and upregulate autophagy in microglial cells in mice. During peptidoglycan infection in the mouse nervous system, enhancing autophagy by adding RAPA could further exacerbate neuronal damage, while inhibiting autophagy had a neuroprotective effect. Studies under different pathological conditions have yielded varying results. A study found that regulating autophagy genes in microglial cells led to a significant decrease in the protein levels of Beclin1 and autophagy-related protein 3 in BV-2 cells treated with LPS (Song et al., 2015). An *in vivo* study revealed autophagy dysfunction in the mouse brain after LPS injection, with significant reductions in Beclin1 and MAP1LC3B-II levels, and increased levels of SQSTM1/p62 in the cortex and hippocampus (Francois et al., 2014). Additionally, it is reported that LPS significantly inhibited microglial cell autophagic flux and the expression of autophagy-related genes by activating toll-like receptor 4 (Lee et al., 2019). The results showed that LPS suppressed autophagy initiation and development, as autophagy activation following LPS treatment was not observed in BV-2 cells. It is noteworthy that the autophagy inhibition by LPS was more pronounced in primary microglial cells compared to BV-2 cells. In the present study, we found that LPS activated the IκB-α/NF-κB pathway in primary microglial cells and increased the expression of pro-inflammatory factors. With an increase in the concentration of LPS, these effects became more pronounced. Furthermore, we pre-treated primary microglial cells with autophagy modulators. It was found that in the LPS group, the expression of p-NF-κB and p-IκB-α proteins increased, while the expression of autophagy proteins Beclin1 and LC3II/I decreased. In the RAPA pre-treatment group, there were enhanced expressions of autophagy proteins and decreased expression of p-NF-κB and p-IκB-α proteins. The results suggest that LPS may exert an autophagy-inhibiting effect through the activation of the NF-κB pathway. Enhancing autophagy may reduce the activation of NF-κB/IκB-α. However, further studies are needed to confirm this.

Autophagy affects many aspects of innate immunity and adaptive immunity. For the human body, autophagy dysfunction can lead to inflammation, autoimmune diseases, or systemic immune disorders (Deretic et al., 2013; Murthy et al., 2014). Microglia, as the intrinsic immune cells of the nervous system, play a crucial role in infectious diseases. Our study found that during the LPS treatment in the primary microglial cells, promoting autophagy effectively reduced the release of pro-inflammatory factors, and suppressed M1 polarization of microglial cells, as evidenced by decreased expression of related cytokines such as IL-1β, IL-6, and CD86, and increased expression of M2 phenotype factors such as Arg-1, IL-10, and CD206. This indicates that autophagy plays a role in regulating cytokine production and M1 polarization of microglial cells during LPS stimulation.

Recent studies have also shown that autophagy plays a role in regulating the production and release of cytokines, inflammatory body activation, antigen presentation, and clearing invasive pathogens (Nakahira et al., 2011; Cadwell, 2016; Munz, 2016). In this study, further *in vivo* experiments in mice demonstrated that LPS resulted in neuronal damage and induced neuronophagocytosis of microglial cells. Inhibiting autophagy with 3-MA further exacerbated neuronal damage, leading to a significant increase in pro-inflammatory cytokines IL-1β and IL-6 levels. Nissl staining showed increased necrotic neurons. To observe the functional status of microglial cells, we stained the CD68, one specific marker of microglial cells, by using immunohistochemistry. We observed a significant aggregation of microglial cells after treatment with autophagy inhibitors, leading to increased structural damage in neural tissues. H&E staining further confirmed increased neuronophagocytosis. Interestingly, enhancing autophagy exerted a neuroprotective effect. By activating autophagy with RAPA, both Western blot and immunofluorescence confirmed increased autophagy. Moreover, compared to the autophagy inhibition group, levels of pro-inflammatory cytokines IL-1β and IL-6 were significantly reduced, necrotic neurons decreased, and neuronophagocytosis by microglial cells was inhibited.

The findings of this study highlight the essential role of autophagy in regulating neuroinflammation, particularly in the context of LPS-induced responses within the central nervous system. Through both *in vivo* and *in vitro* experiments, we established that LPS significantly promoted M1 polarization of microglial cells while concurrently triggering pro-inflammatory cytokine release, contributing to neuronal injury. Importantly, our results revealed that enhancing autophagy using RAPA had a protective effect by counteracting LPS-induced neuroinflammation. Furthermore, the inhibition of autophagy via 3-MA not only facilitated increased inflammatory responses but also led to enhanced neuronophagocytosis and subsequent neuronal damage. These findings highlight the potential of autophagy modulation as a promising therapeutic approach for alleviating neuroinflammatory conditions and preserving neuronal health. Future research should focus on elucidating specific autophagy pathways and their interactions with immune signaling mechanisms, which may further refine therapeutic strategies targeting autophagy in neurodegenerative disorders and other inflammatory diseases affecting the central nervous system.

## Data Availability

The raw data supporting the conclusions of this article will be made available by the authors, without undue reservation.
